# Engaging Faith-Based Organizations for Promoting the Uptake of COVID-19 Vaccine in India: A Case Study of a Multi-Faith Society

**DOI:** 10.3390/vaccines11040837

**Published:** 2023-04-13

**Authors:** Gopal Krishna Soni, Anumegha Bhatnagar, Anil Gupta, Amrita Kumari, Sonal Arora, Surbhi Seth, Apurva Rastogi, Natasha Kanagat, Rebecca Fields

**Affiliations:** 1 John Snow India Pvt. Ltd. (JSIPL)-Plot No. 5 & 6, First Floor Allied House, Pocket 10, Sector B, Vasant Kunj, Delhi 110070, India; 2 John Snow Inc., 2733 Crystal Drive, 4th Floor, Arlington, VA 22202, USA

**Keywords:** vaccination, vaccine inequity, faith-based partnerships, faith-based organizations, FBO, community engagement, strategic collaboration for COVID-19 vaccination, vaccine hesitancy

## Abstract

Mass vaccination, currently the most promising solution to contain communicable diseases, including COVID-19 requires collaboration between a variety of partners to improve the supply and demand and alleviate vaccine inequity. Vaccine hesitancy features in WHO’s list of top 10 threats to global health, and there is plethora of disinformation instigating conflict between COVID-19 vaccination drive and religious sentiments. Negotiating public health partnerships with FBOs (Faith Based Organizations) has always been challenging. A handful of faith leaders have always shown resistance to ideas such as child immunization, and family planning. Many others have been supportive on other fronts like helping people with food, shelter, and medical aid in the times of public health crisis. Religion is an important part of life for the majority of the Indian population. People confide in faith-based leaders in the times of difficulty. This article presents experiences from the strategic engagement with FBOs (entities dedicated to specific religious identities, often including a social or moral component) to promote uptake of COVID-19 vaccination, especially among the vulnerable and marginalized communities. The project team collaborated with 18 FBOs and more than 400 religious institutions to promote COVID-19 vaccination and build confidence for the vaccination program. As a result, a sustainable network of sensitized FBOs from diverse faiths was created. The FBOs mobilized and facilitated vaccination of 0.41 million beneficiaries under the project.

## 1. Introduction: Vaccine Hesitancy

Vaccination continues to be the most effective method for containing the spread of communicable diseases and limiting damage by developing herd immunity, especially the ones such as COVID-19 [1]. Their efficacy and safety, however, have been frequently challenged by a variety of persons and groups claiming religious, scientific, and political reasons [2]. According to the World Health Organization, vaccine hesitancy is the unwillingness or inability to take vaccinations despite the availability of vaccines [3]. Vaccine hesitancy has been identified as a major impediment to achieving optimal disease control and elimination by numerous public health initiatives [4]. Despite the scientific consensus that vaccines are beneficial to public health, there are still differences in the acceptance level among individuals and communities. Vaccine hesitancy is complex, has various levels, and varies with time and circumstances. Galagali, Kinikar and Kumar (2022) in their ‘Hesitancy determinant matrix’ have threaded the factors influencing the behavioral choice to accept, postpone, or refuse vaccines under three heads: (1) contextual influences, (2) individual and group influences, and (3) vaccine/vaccination-specific issues [5].

The contextual factors comprise influences arising from political, socio-cultural, historic, environmental, economic, and institutional factors. ‘Religion’ and ‘culture’ are integrated within the contextual factors. The individual/group influences comprise of awareness, knowledge, beliefs, and attitudes. Vaccine-specific issues contain factors such as questions around the introduction of new vaccines, cost, risk/benefits, operational issues, the strength of recommendation, schedule etc. The interplay of these factors has a major role in determining hesitancy or confidence in the vaccine. Over the last decade, the dynamics of these factors have changed substantially. COVID-19 vaccine with a status “approval for restricted use in an emergency situation” faced an unprecedented level of ‘Vaccine Hesitancy’ offered by a complex interplay of the above-mentioned factors.

India’s massive COVID-19 immunization program began on 16 January 2021, and it was one of the largest in the world. The program started with two indigenously developed vaccines- Covishield (Oxford-AstraZeneca/Serum Institute of India) and Covaxin (Bharat Biotech/Indian Council of Medical Research). It gradually included vaccines like Corbevax, Zycov D, Sputnik [6]. The country followed a graded roll-out model, prioritizing those with maximum exposure to the virus such as health workers and frontline workers. Later, the elderly population (people above 60 years of age) and those with co-morbidities were included in the eligibility criteria. This was followed by extending the scope of vaccination to eligible beneficiaries above 45 years and then gradually to younger age groups. [7] In May 2021, the vaccination target was set at covering 0.93 billion adult population (i.e., 18 years and above) by the end of 2021. However, by September 2021, only 13.73% of the target population had received the COVID-19 vaccine [8]. Although the Government did account for vaccine hesitancy as a potential barrier evident in the “COVID-19 Vaccine Communication Strategy” by MoHFW [9], vaccine hesitancy emerged stronger than it appeared initially. The spread of mis/disinformation multiplied with the increased use of social media and the internet giving less bandwidth for policy to respond. Vaccine hesitancy being complicated and multifaceted requires non-traditional solutions [10]. To address the challenge presented by vaccine hesitancy, a communication strategy involving Faith Based Organizations (FBOs) and leaders was devised. FBOs are groups that promote distinct religious identities, sometimes with a social or moral component. They may be congregations; national networks, which include national denominations, their social service arms (for example, Catholic Charities, Lutheran Social Services, Temple and mosque cooperative societies), and networks of related organisations (such as Young Men’s Christian Association); and freestanding religious organisations, which are incorporated separately from congregations and national networks [11].

## 2. Context: Overcoming Obstacles to Vaccine Uptake by Jointly Developing Solutions with Faith Leaders

Religious faith represents a key social and environmental driver that can influence an individual’s beliefs, health behavior, and practices [12]. Health promotion programs rooted in the involvement of faith-based organizations and the engagement of faith leaders as advocates for disease prevention and treatment have proven effective in bringing about positive change in community health [13]. Faith-based organizations bridge the gap between publicly funded healthcare facilities and the needs of individuals who cannot access public healthcare due to poverty and lack of access in rural settings [14]. In times of crisis such as the global COVID-19 pandemic, the role of FBOs has been critical in controlling the spread of the virus and vaccinating the population to curb illness and fatalities due to the disease.

Faith and immunization have historically had both positive and negative associations. While some religious leaders took charge in pushing vaccination, others needed a lot of persuasion to agree to and tolerate this strategy [15]. Communication strategies centered around faith leaders have led to favorable outcomes in tackling misinformation and reducing the spread of infectious diseases, including poliomyelitis (polio), human immunodeficiency virus (HIV), and Ebola [16]. A successful example is the Polio Eradication Initiative which involved the participation of faith leaders in polio campaigns to enhance public confidence leading to an increase in vaccine coverage [17].

Vaccine hesitancy has been identified among the top ten threats to global health by the World Health Organization [18]. Hesitancy toward the newly developed COVID-19 vaccines is also evident across the world [19]. Vaccine hesitancy is defined as delay or refusal to accept or refuse immunization despite the availability of vaccination services [20]. In India, a significant proportion of the eligible population is hesitant toward COVID-19 vaccines [21]. A deeper understanding of nuanced community-level factors driving vaccine hesitancy, along with identifying trusted sources for decision-making is essential to formulate the strategy to increase uptake and address barriers to vaccination [22]. Faith leaders are often considered a trusted resource in the community, as they influence health behavior at individual and socio-cultural levels [23]. Faith leaders are members of the community who are recognized for their role in guiding and inspiring religious and faith-based beliefs and practices of the community, playing an authoritative and influential leadership role. Faith leaders and organizations represent a key resource in building vaccine confidence in the community, by promoting messages of best practices with regard to vaccines. In this article, we discuss the contributions of Faith-Based Organizations (FBOs), in mobilizing the vulnerable and marginalized for COVID-19 vaccination. The article shows how effective communication with faith-based communities can significantly improve the outcomes of public health programs. Lessons from case studies shared in the article can be generalized into a set of policy suggestions for improving future public health projects globally because the mentioned concepts apply to various scenarios.

## 3. Materials and Methods

When COVID-19 vaccination efforts began, utilizing all possible modes of communication became crucial for informing the public appropriately and obtaining effective results. The majority of the Indian population places a high value on their FBOs. As a result, we examined how the involvement of FBOs contributes to community engagement in the time of public health emergencies.

Although the project activities span 18 prominent states in India, collaboration with faith-based organizations was eminently considered in the project states representing religious minorities and areas showing higher resistance to vaccination due to faith-based reasons. The selection of FBOs was based on (1) the size of the followership group represented by the FBO (2) display of community ownership in the time of public health crisis (3) the capacity and resource pool to facilitate the vaccination and, (4) the willingness to engage in strategic collaboration. The level of engagement of the FBO was closely observed for the width of impact and depth of community response on tangible and intangible parameters. The identification and engagement of FBOs included line listing of FBOs registered in the project geography, comparing the FBOs on the size of follower group by factoring in the population of devotee’s basis religion, caste etc., identifying vaccination champions from FBOs who could be recognized for supporting COVID-19 vaccination, creating a sustainable network of FBOs duly sensitized and oriented on COVID-19 immunization by building capacities of FBOs on COVID-19 virus and vaccination through sensitization meetings. The details of the process are shared in the forthcoming sections.

## 4. Operational Framework

**Figure 1:**
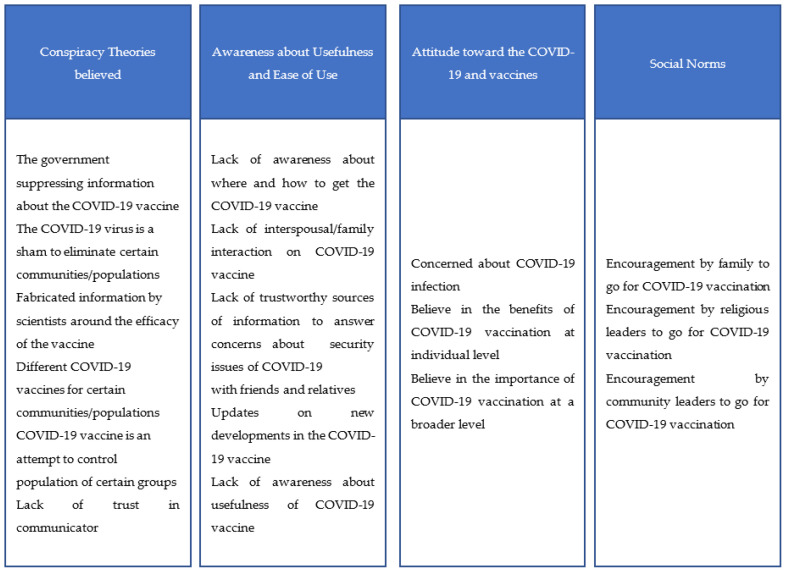
Factors influencing acceptance of COVID-19 vaccine.

A scoping review by Joshi et al. [24] found “Trust in authorities” as an important determinant of vaccine acceptance. Misinformation reaching through various sources could have an effect on the trust in authority, vaccine safety, and efficacy [25]. Governments and societies must gauge current levels of willingness to receive COVID-19 vaccines at the community level. Intervention models to improve vaccine literacy and acceptance should directly take up community-specific concerns, and misconceptions, and be sensitive to religious or cultural beliefs. A few studies confirm the nerve centers of misinformation can be controlled with the help of organizations trusted in specific communities or areas [26,27]. Faith-based organizations have played a significant role in community engagement. The project team believed that co-creating interventions with faith leaders, who are often the gatekeepers of tight-knit communities, can lead to the creation of positive messages about the COVID-19 vaccine that people in the community can relate to. This makes people more confident in vaccines. Akther and Nur (2022) synthesized behavioral theories with COVID-19 acceptance factors to create a COVID-19 vaccine acceptance model [28]. The Behavioral and Social Drivers (BeSD) model by the World Health Organization also includes religious and community leader norms as important elements of COVID-19 vaccine acceptance [29]. Figure 1 lists factors collated from the above-mentioned models. FBOs have a role to play in creating awareness, negating misinformation, resolving doubts, and creating a positive environment for acceptance of the COVID-19 vaccine.

**Figure 2:**
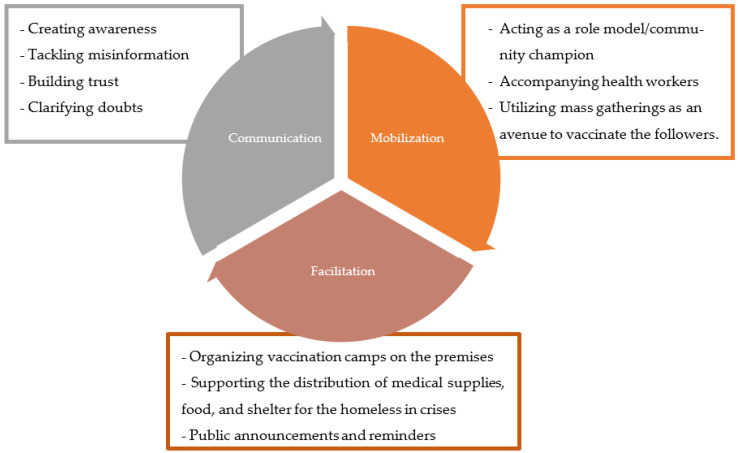
Operational Framework of Community Engagement through Faith-Based Organizations (FBOs).

Because FBOs are a crucial resource in helping the community develop vaccine trust, we designed the community engagement and mobilization approach to make FBOs frontrunners in areas with a high inclination towards religion and faith and low confidence in the vaccination drive. The key implementation strategies used to achieve community engagement goals were based on an operational framework depicted in Figure 2.

The collaboration with FBOs aimed to achieve two goals, garnering community response on the COVID-19 vaccine and developing long-term relationships with the FBO to ensure future readiness by developing trust. In the short run, the influence of religious leaders was utilized to garner support for COVID-19 vaccination and build confidence in vulnerable communities by highlighting the vaccine’s safety and efficacy. The FBOs also helped to counter myths/rumors associated with religious beliefs by preaching the importance of preventive care during their recitation in religious gatherings. FBOs led the creation of an enabling environment for the COVID-19 vaccination program with a focus on vulnerable communities.

The first step in this direction was to partner with faith-based leaders who could influence the community. Merging a science-based approach with a faith-based outreach was essential to persuade those who were hesitant purely based on a religious perspective. Secondly, the physical manifestations of faith-based organizations like churches, mosques, temples, gurudwaras (place of worship for Sikhs) etc., have always been the haven of service provision during emergencies. During the COVID-19 crisis, these institutions had become a critical source of food, medical resources and sometimes shelter. Therefore, strategically partnering with these institutions to reach the marginalized living in the close vicinity of these institutions is important to reach the last mile. After making forays into the community with the help of the FBO leaders, the project team forged strategic relationships with the community leaders. Beyond the current project efforts, the goal was to create lasting ownership of not only this vaccination effort but any future public health crises which may arise.

The FBO leaders were historically known for being role models for their followers, making it critical that these leaders become advocates and voices of their communities. In times of crisis, the community may not accept the word of political or social leaders but many will follow the guidance of their faith leaders. Therefore, it was strategically important to ensure that the FBO leaders understood the efficacy, safety, and benefits of the vaccine and also became long-term change agents in communities for public health interventions.

Implementation process: The project adopted a list of strategic activities to achieve its goals and objectives.

- Line listing of FBOs in the project geography which could influence the target communities
- Identifying vaccination champions from FBOs who could be recognized for supporting COVID-19 vaccination
- Creating a sustainable network of FBOs duly sensitized and oriented on COVID-19 vaccination
- Building capacities of FBOs on SARS coV-2 virus that causes COVID-19, and on vaccination through sensitization meetings with religious leaders to clear myths and misconceptions related to COVID-19 vaccination
- Dissemination of written appeal/video snippets from key religious leaders in the support of COVID-19 vaccination
- Targeting vulnerable populations in project areas by the intense use of social media along with mass media and mid-media with appeals of FBO leaders.
- Dispelling rumours related to vaccination through announcements and appeals during religious meetings/gatherings
- Organizing COVID-19 vaccination sessions within FBO premises to increase vaccine confidence in the target communities
- Leveraging mass gathering at religious places for vaccination such as Friday prayers, EID gatherings in the mosques and Sunday masses, and Christmas campaigns in the churches.

### 4.1. Communication

The project team collaborated with various FBOs in the project states. Sensitization meetings and training sessions were done with Muslim institutions in the states of Maharashtra, Madhya Pradesh, Jharkhand, Rajasthan, and Jammu & Kashmir (For example, Jamiat-Ulema e Hind in Rajasthan, and Tajul Masjid in Bhopal). A positive environment for vaccination emerged from extensive community engagement activities, such as advocacy meetings, and announcements from prominent mosques during Eid prayers and Friday prayers. Written appeals and video snippets from the leaders were spread through social media platforms such as Facebook and WhatsApp. Live streaming of sermons at religious events was done on Facebook, and vaccination camp-related information was disseminated through WhatsApp. Community ownership of the vaccination program was developed through the FBOs. The leaders were oriented on COVID-19 appropriate behaviors compatible with religious teachings. COVID-19 vaccination was done during these events. These activities have also reduced vaccine hesitancy and increased vaccine confidence among community members who had reservations about vaccination.

### 4.2. Facilitation

The project team collaborated with the largest Sikh institution, the Sikh Gurudwara Prabandhak Committee, and organizations like Dhan Dhan Satguru and Radha Swami Satsang in Punjab. As a result, vaccination camps were held on the grounds of Gurudwaras and other Faith-based organizations, and more than one hundred thousand beneficiaries were immunized on the grounds of FBOs in the state. The project collaborated with churches affiliated with different states in the northeastern states such as Nagaland, Manipur, Mizoram, Assam, Meghalaya, Tripura and Arunachal Pradesh. In Nagaland, special COVID-19 vaccination clinics were organized on Sundays. As part of this campaign, termed Sunday vaccination or the Har Church Dastak Initiative (Knock every church for COVID-19 vaccination), the pastors made announcements during the Sunday Masses before the devotees were immunized. The Vaccination camps were also conducted in collaboration with several Hindu FBOs in various project states.

### 4.3. Mobilization

The team also carried out COVID-19 vaccination awareness camps in well-known FBO premises. The devotees and pilgrims were called upon by the faith leaders to attend the awareness cum vaccination. The project held awareness sessions to educate people about the importance of vaccination and resolve doubts of reluctant devotees and pilgrims. Along with this, teams have also utilized religious occasions like Jagrans, religious fairs, and other religious gatherings to create awareness and vaccination in these gatherings. Additionally, in the States of Jharkhand and Maharashtra, on-ground mobilization was conducted to address vaccine hesitancy, banners were displayed during Eid prayers, counseling sessions were hosted after prayer meetings and religious leaders were also sensitized about the importance of getting fully vaccinated. In Jharkhand, 50 faith leaders took the COVID-19 vaccine during the Eid gatherings to act as role models. Similar efforts were undertaken to motivate faith-based leaders to act as role models. Over the ensuing eighteen months since August 2021, the strategic engagement took shape in the form of sensitizing key religious leaders, using their influence to show support for the COVID-19 vaccination, dispelling rumors and myths, leveraging their reach to create an enabling environment for the vaccination, and utilizing the places of worship for information dissemination and administering vaccinations.

## 5. Results

The immediate impact of engaging faith leaders and faith-based organizations was vaccinating 0.41 million beneficiaries of eligible cohorts till January 2023. This was done through awareness campaigns that sensitized many FBO leaders and their followers on the efficacy and safety of the vaccines. FBOs were made aware of the COVID-19 vaccination program and encouraged to motivate their congregations to get vaccines and protect themselves against the COVID-19 disease. COVID-19 preventive behaviors (wearing masks, social distancing, and maintaining hand hygiene) were adopted by faith-based leaders publicly. Compliance with COVID-19 appropriate behavior and vaccination practice were emphasized at worship places. Further, communication within families to create a more supportive environment for the COVID-19 vaccination for all eligible beneficiaries was facilitated. Reminding families about the responsibility of every follower to get himself/herself vaccinated as well as act as a “Cause Ambassador” in his/her area of influence was ensured.

**Figure 3:**
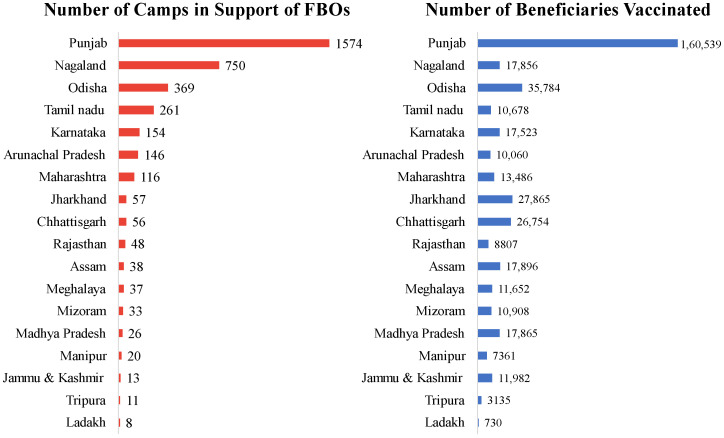
State-wise results achieved by FBO engagement. Total Camps: 3717, Number of beneficiaries: 410,881.

The engagement of faith leaders helped disseminate positive messaging, thereby increasing confidence in vaccines and reducing vaccine hesitancy. In the geography of 298 districts across 18 states of India, 3717 camps were organized with the support of FBOs, which included mobilization efforts, the presence of local FBO leaders, and conducting the session in the premises of the religious practice (Figure 3).

The engagement efforts are also expected to bring community level acceptance to vaccination in the long run. Some efforts were initiated by project team such as the creation of support groups to facilitate an enabling environment for the vaccination which can act as behavioral drivers to create a positive atmosphere for vaccination among vulnerable and marginalized communities. With the concerted efforts of the project team in partnership with the FBOs across the 18 states and union territories enrolled in this project, thousands more continue to get fully vaccinated and protected every day. Some selected case studies of this work from various geographies are:

### 5.1. Case Study from Mansa, Punjab State

Radha Soami Satsang, Beas and FBO based in the Mansa district of Punjab became a key partner in reaching the community in the village of Jhunir. This remote village is underserved in many aspects, however, the population residing here has great faith in this religious institution. The leaders of the FBO have backed the immunization program and have made announcements from their institutions to encourage the community to get the COVID-19 vaccine. Before holding a vaccination camp, the team conducted group/interpersonal communication activities and distributed flyers to dispel myths about COVID-19 vaccination among the village population. The immunization campaign was a huge success, and over 600 people were vaccinated through the Radha Soami Satsang, Beas partnership.

### 5.2. Case Study from Falihamari Habi Char, Morigaon, Assam

In a geographically and religiously diverse country like India, the barriers to access healthcare or other social care services can be multi-layered as seen in the case of Falihamari Habi Char in Assam. Separated from the mainland by the river Brahmaputra, the residents of the area, who are predominantly farmers, have to walk miles to access most services. There is little to no public transportation and the population is cut-off from most public services. For COVID-19 vaccination, there was minimal response and most community members were wary of a largely unknown entity administering vaccination. However, the strategic intervention utilized in other regions proved effective here by engaging the Imam (religious leader of the mosque) as an ally to the vaccination efforts. Critical interventions were implemented by the Imam such as announcements and counselling sessions at the mosque itself after the Friday prayers and shifting the base camp for vaccinations within the char area so the community members could easily access it without losing a day of wages travelling to the mainland.

### 5.3. Case Study on Eid as an Awareness Campaign

In states across the country, engagement with mosques on the occasion of Eid to spread the message that vaccines are essential “to celebrate the festival with uninterrupted joy” has helped immensely to improve COVID-19 vaccine uptake. Social media posts and printed materials in local languages and dialects were disseminated throughout the festive season. In Jharkhand and Maharashtra, on-ground mobilization was conducted to address vaccine hesitancy, banners were displayed during Eid prayers, counselling sessions were hosted after prayer meetings and religious leaders were sensitized about the importance of getting fully vaccinated. Special COVID-19 vaccination camps on Eid at various sites of the celebrations in Maharashtra which reached over 500 devotees. In Aurangabad, public addresses through miking on vans and street plays were organized to inform and counsel those participating in the Eid festivities. The religious and community leaders were oriented on both COVID-19-appropriate behaviors for practicing the faith safely and on the benefits and efficacy of the vaccine. Hundreds were reached through this messaging and were able to overcome their vaccine hesitancy. The most significant long-term outcome has been the ownership of the vaccination program by faith-based leaders. This will ensure the sustained participation of the FBOs in the current and future public health crises and vaccination drives.

### 5.4. Case Study on Hemis Changchun Chosling Committee of Buddhist Monks in Ladakh

Buddhism is the most practiced religion in Ladakh and the Monastery plays an important role in determining the behavior pattern of the community. Understanding the key role of these institutions, the project team approached the HCC Committee (religious group of the Buddhist Monastery) located at Hemis Gompa, which is the one prime body having a strong influence over the people of Ladakh. The committee prohibits the organization of any event other than a religious event on the institution’s property. The project team consistently endeavored to organize monks’ meetings for COVID-19 vaccine sensitization. The team interacted with the major leaders at the seminary, who permitted them to organize the awareness session. Around 50 committee members participated in this meeting and initiated the communication efforts to help and encourage the villagers in their areas to take the COVID-19 vaccine.

## 6. Discussion

This study highlights the significance of partnering with FBOs and religious leaders in vaccination programs to engage communities meaningfully by dispelling myths, clearing up misunderstandings, building vaccine confidence, and encouraging vaccine uptake. Our findings echo the results of a systematic review by Syed et al. [30] who emphasizes the effectiveness of FBOs in supporting vaccination efforts globally because FBOs have deep relationships and mutual trust with communities which can be harnessed to help communities feel safe and confident in choosing vaccinations. Syed and co-authors encourage the public health community to intentionally engage FBOs more frequently for sustained program effectiveness and to close the vaccination gap in underserved communities. The PRRI–IFYC Religion and the Vaccine Survey in the US also suggested similar findings [31]. However, while some research in the US and Pakistan suggests that religious leaders may influence communities not to get vaccinated due to their own vaccine hesitancy [32,33] most research across several countries still advocates for greater FBO engagement [34,35]. Moreover, our study also suggests that hyper-local efforts that take into account the local dialects and beliefs are going to be critical in sustaining vaccination efforts especially as we pivot back to focusing on routine immunization, coverage of which has slipped significantly during the COVID-19 pandemic [36].

Right from the beginning of the COVID-19 crisis, international, national, state, and local organizations have looked towards faith-based organizations for support. In May 2020, the World Health Organization (WHO) and UNICEF brought together a panel of state government officials and leaders of FBOs such as Catholic Faith, Art of Living, Jamaat-I-Islami Hind, All India Muslim Personal Law Board, Jamiat-e-Ulema-Hind, All India Ulema Council, ISKCON, Isha Foundation, Rama Krishna Mission Math etc. The state government ministers appealed to the religious leaders to raise their voices against stigma and discrimination by sharing powerful examples from religious scriptures through their network and giving a voice to the unseen ‘Corona Heroes’ in the field. The religious institutions were also requested to provide quarantine facilities to support the government’s efforts.

To maximize the efforts, the project team deemed engagement with FBOs as one of the most strategically important interventions. Every previous vaccination effort such as for polio or measles-rubella had proven that communities across socioeconomic barriers are often held together by their religious faith. Finding allies within faith-based leaders was essential to reach the hardest to reach and most vulnerable. The local FBO and their leaders mostly spoke the dialects of the community they were engaged with and had the complete trust of the families. Often very personal decisions about marriage, childbirth, healthcare, and social engagements are made in consultation with local religious leaders.

Many valuable lessons were learned on targeting devotees with the help of faith-based leaders during key religious and cultural festivals throughout the year. Sustaining the gains is much more important than the immediate results and this can be ensured by:

1. Connecting with religious leaders to create a movement to support vaccination and motivate and educate followers to adopt other healthy behaviors that are compatible with religious teachings
2. Establish COVID-19 support groups in the community
3. Long-term participation of FBOs in grassroots mobilization and tackling vaccine hesitancy related to faith-based concerns
4. Religious sites such as temples, mosques, gurudwaras, etc. established as having a key role in vaccination drives
5. Establish at least one state and district-level partnership with FBOs to enhance ownership of vaccination programs by FBOs

Faith-based organizations (FBOs) in India have historically been part of the vaccination efforts of the government [37,38]. In prior vaccination drives such as for polio and Measles-Rubella, the FBOs had a significant role in guiding vaccine confidence, promoting healthy behaviors, and providing physical safe spaces to both address concerns of their congregation and run vaccination camps from churches, temples, gurudwaras, and mosques. For example, in 2018, the religious leaders in Godhra joined hands with private doctors to ensure that the vaccine was administered to children from the minority community in the town [39]. In a student population of 2800, only 110 had been vaccinated. The parents were apprehensive about the vaccine both for medical and religious reasons. The school organized a function with the support of the local religious leader and district development officer who helped dispel myths and rumors about the vaccine and encouraged students to get vaccinated at the spot. Similarly, in UP, the polio vaccination efforts by UNICEF relied heavily on the religious leaders and teachers at Madrasas (Madrasas are type of school or college that are the nucleus of the cultural and educational life of Muslims) over a period of 8 years to reduce vaccine hesitancy [40]. The program’s success can be gauged by the fact that at many places, polio vaccination booths were set up in and around the premises of mosques and Madrasas. Mosque announcements were made to the community regarding the importance of the vaccine and immunization for children. This was an unimaginable scenario earlier and perhaps played a significant role in changing the perception of the minority community with regard to polio vaccination. Partnerships have also been formed under the M-RITE project with a number of FBOs in different states, such as the Sikh Gurudwara Prabandhak Samiti (SGPC), a prominent Sikh religious organization in Punjab. The SGPC made an appeal in favour of vaccination and assisted in spreading awareness and administering vaccinations within the Golden Temple grounds in Amritsar. Similarly, Tajjul-Masjid, Asia’s largest mosque, held an awareness campaign and administered vaccinations within its grounds in front of nearly 200 religious’ leaders, who pleaded for assistance in educating the public about the need to dispel myths and misconceptions and the advantages of the COVID-19 vaccination.

There are many instances of leveraging support from FBOs to promote vaccination in India, especially in the last two decades. FBOs acted as a vital cog in the wheel of involving all stakeholders in reaching the last mile and vaccinating every beneficiary. While for polio and measles campaigns the beneficiary group was primarily limited to children, in the case of COVID-19 it was to vaccinate all age groups above 12 years. Due to the sheer number of beneficiaries, it required an enormous effort. Even after mitigating the technical challenges of supply chain management to ensure vaccine availability, geographical access and effective communication remained the task to convince an adult free-thinking population diverse in ethnicity, religious belief, religion of the need for vaccination. FBOs acted in unison with the local community leaders, political leaders and acted as a trigger for the vaccination which ensured success of COVID-19 vaccination program in India.

The expanding transnational significance of FBOs cannot be denied either. FBOs are receiving more scholarly and policy attention at International Organizations such as United Nations (the juncture of Global public policy). A noteworthy number of NGOs with consultative status with the UN’s Economic and Social Council (ECOSOC) are FBOs [41]. The relationship between religion and health has been evidenced by the role of religion in the response to the 2014–2016 Ebola outbreak and the global HIV epidemic [42,43]. The case examples shared in the current article signify that inculcating a sense of ownership among FBOs and their leaders is essential for the success of programs. Based on project experience, we learned that most FBOs are open to collaborating with a wide range of allies as long as they share ideological preferences and goals. There are a large number of FBOs that are operating at the international level such as Aga Khan Foundation, Buddhist Global Relief, Catholic Relief Services etc. that have done demonstrable work in the health and development sector worldwide. The smaller but local FBOs have a reputation with local communities and can contribute immensely to the health and other development programs if bestowed with ownership and matching ideologies on development.

## 7. Conclusions

The role of FBOs was critical in addressing myths especially related to faith/beliefs, building confidence among community members thereby leading to increased acceptance of COVID-19 vaccine and behavior change in communities. The influence of FBOs can be leveraged in adult vaccination including routine immunization in children. The FBOs encouraged the community to abide by official directives and backed national measures in religious sensitive areas. The team also took an active position against false information regarding the pandemic and the nationwide immunization program. Additional methods for adjusting religious services were also adopted. Some of the organizations facilitated vaccination camps and offered premises for medical camps to serve critically ill cases. This shows that a dedicated effort will have a far-reaching impact on community engagement. We see FBOs as a direct bridge with community. Partnerships with them might even become more significant over time, particularly for routine immunization and life course vaccination.

It makes sense that the public health and FBOs representing hard-to-reach communities would want to collaborate with one another to further the shared objective of preventing suffering. Each side has vital content that the other does not, therefore neither can afford to move forward without the other. A careful and rational approach to cooperation is essential for the successful resolution of vaccine hesitancy.

## 8. Recommendations

In collaboration with public health stakeholders, FBOs have a significant role to play. In light of the aforementioned experiences, we share the following recommendations:

### 8.1. Recommendations for the Practitioners

We Recommendations Working at Three Levels:

Upgrading roles: In India, faith leaders have been active in the political landscape so much so that a few ministerial roles have been served by a few of them. The faith-based leaders are already playing a dual role, promoting spirituality and inculcating the healthy lifestyle, sensibility to towards environment and humanity. Some have gone far to the extent of running charitable hospitals, schools, and shelters for the needy. Having done all this, they deserve a role more than working as mouthpiece on COVID-19 communications. They must be invited to take leadership roles, particularly those representing marginalized communities and religious minorities.

Integration: Faith-based leaders are among the most powerful community partners. Integrating FBOs in the public health system will ensure that actions respect cultural differences and don’t clash with deeply held (religious) values. Moreover, their presence in conflict-reducing. The mistrust on Government and public health agencies whenever instigated through misinformation can be lessened with the help of FBOs. The limitations of what is currently known about safety, efficacy, breakthrough cases, variations, and other matters that could worry the general public can be conveyed by utilizing the expertise of FBOs.

Operational partnership: Deep rooted FBOs with motivated taskforce and unconditional followership and well-developed premises offer a lucrative advantage for premises and taskforce for vaccine storage, distribution and inoculation. They are accessible and trustworthy institutions for the local devotees and therefore, important for the existing and potential health programs. Such collaborations ought to continue after the COVID-19 pandemic and constitute an ongoing component of the community.

### 8.2. Recommendations for the Researchers

The collaboration follows the basics of existing behavior change theories and frameworks on behavioral and social drivers for vaccine uptake and offers a bedrock to test and upgrade the frameworks. The vaccine hesitancy models can be can be further tested by including mediating or moderating variables such as religion, faith, beliefs, age, gender, and so forth. This will help gain a thorough understanding and exploration of the other influential factors to direct policymakers and protect human lives.

## 9. Strengths and Limitations

### 9.1. Strengths

Our article draws on a diverse set of experiences in engaging with FBOs from different religions in almost 20 states in India. This extensive scope offers us detailed insights into how best to partner with FBOs in different contexts. Another strength of this article is that all the activities described were implemented over several months affording us time to implement and adapt our FBO-community engagement plans and improve vaccination coverage. Faith leaders are members of the community who are acknowledged for their role in directing and motivating religious and faith-based beliefs and practices of the community, acting in an influential leadership capacity. By disseminating messages about vaccine best practices, religious leaders and organizations serve as an important resource in helping the community develop its vaccine confidence.

This approach enables behavior change in the project/program to address the barriers entrenched in sociocultural and religious beliefs. Vaccine support messaging and information coming from a faith-based leader will resonate and is seems valid to utilize. The results of public health programs can be greatly enhanced by effective communication with faith-based communities.

### 9.2. Limitations

The article reports the experiential insights of collaboration with FBOs at the ground level for the implementation of a National program. However, our approach was ad-hoc when it came to identifying and approaching the FBOs for collaboration. We recommend that other projects that seek to engage FBOs identify criteria by which to select the organizations so that they can serve as a blueprint for other similar initiatives. Our approach to measurement was also more anecdotal and we suggest future projects devise outcomes-based research that clearly links FBO engagement to improved health impact. Most of the Faith-Based leaders except “Brahma Kumaris” (a spiritual movement in India known for the prominent role played by women) were men. Moving forward, it would be advantageous to investigate and facilitate the participation of female faith leaders to invite higher female participation and resolution of gender-specific issues.

At the field level following limitations were realized which can be managed with careful planning in the future:

- Some groups could not be mobilized despite being approached through FBOs. They had deeper apprehensions that required efforts at a personal level rather than a mass approach. Although not a significant number some of the faith leaders themselves were not convinced about vaccination even after the orientation and their own resistance might affect the behavioral pattern of the community
- Despite repetitive efforts, the project team could not reach or engage a few prestigious religious bodies with strong influence in the community.
- Suboptimal ownership of the few faith leaders in the program because of their indifferent opinions was also evident in a few low-performing areas.
- Working with FBOs requires efforts and multiple meetings for sensitization and securing their buy-in for the cause. Some religious leaders after intensive persuasion efforts do not wish to be associated with the cause. The project in that case will then need to identify other community-based leaders to further the cause.

## Data Availability

No new data were created or analyzed in this study. Data sharing is not applicable to this article.
